# Visual Outcomes of Traumatic Lens Dislocations and Subluxations Managed by Pars Plana Vitrectomy and Lensectomy

**DOI:** 10.3390/jcm12226981

**Published:** 2023-11-08

**Authors:** Mădălina-Claudia Hapca, George-Adrian Muntean, Iulia-Andrada Nemeș-Drăgan, Ștefan Cristian Vesa, Simona-Delia Nicoară

**Affiliations:** 1 Doctoral School of Medicine, Iuliu Hațieganu University of Medicine and Pharmacy, V. Babeș Str. 8, 400012 Cluj-Napoca, Romania; georgemuntean99@gmail.com; 2 Ophthalmology Clinic, Emergency County Hospital, 3–5 Clinicilor Str., 400006 Cluj-Napoca, Romania; d_iulia_a@yahoo.com; 3 Department of Ophthalmology, “Iuliu Hațieganu” University of Medicine and Pharmacy, V. Babeș Str. 8, 400012 Cluj-Napoca, Romania; 4 Department of Pharmacology, Toxicology and Clinical Pharmacology, Iuliu Hațieganu University of Medicine and Pharmacy, 400012 Cluj-Napoca, Romania; stefanvesa@gmail.com

**Keywords:** lens dislocation, subluxation, pars plana vitrectomy, pars plana lensectomy, eye trauma

## Abstract

Aim: The aim of this study was to evaluate the visual outcome of lens dislocation and subluxation managed by pars plana vitrectomy (PPV) and lensectomy in patients with open (OGIs) or closed globe injuries (CGIs). Methods: Medical records of 70 consecutive patients treated by PPV and lensectomy over a period of 11 years (1 January 2010–31 December 2020) were retrospectively reviewed. We collected demographic data, best corrected visual acuity (BCVA) using a Snellen Chart pre- and postoperatively, associated ocular injuries and treatment strategy. Visual outcome was evaluated according to the final BCVA which was defined as poor <0.1 or good ≥0.1. Results: The mean age was 57.9 ± 17.6 years. CGIs were present in 49 (70.0%) cases and open OGIs in 21 (30.0%) cases. The dislocation mechanism was zonular lysis in 59 cases (84.3%) and capsular rupture in 11 cases (15.7%). The intraocular lens implant (IOL) was sutured to the sclera in 51 (72.9%) cases or positioned in the capsular bag or in the sulcus in 3 (4.3%) cases and 1 (1.4%) case, respectively, whereas 15 (21.4%) patients remained aphakic. A good BCVA ≥ 0.1 was achieved in 45.71% of the eyes. The presence of retinal detachment (RD) (*p* = 0.014), iridodonesis (*p* = 0.011) and initial BCVA (*p* = 0.000) achieved statistical significance in predicting visual outcome. After treatment, 45.71% of patients achieved a final BCVA ≥ 0.1. Conclusion: RD, iridodonesis and initial BCVA were risk factors for poor visual outcome in our series.

## 1. Introduction

Ocular trauma is one of the most common causes of unilateral blindness with a peak incidence in early adulthood and in the sixth and seventh decades of life [1]. An estimated 55 million ocular injuries occur annually, resulting in 1.6 million cases of blindness, 2.3 million cases of low vision and nearly 19 million cases of monocular blindness [2]. Lens dislocation and subluxation occur when the zonular fibers which anchor the lens to the ciliary body are ruptured in the context of open or closed globe trauma [3]. These fibers play a special role in suspending and holding the lens in place inside the eye. In penetrating and perforating eye injuries, the zonular fibers can break through a direct or indirect mechanism, whereas in blunt trauma, the zonular fibers are stretched or ruptured due to an equatorial expansion secondary to anterior–posterior compression of the globe [4]. While an isolated lens pathology carries a very good prognosis, the final visual outcome in patients with trauma is usually poorer because of the associated lesions [5]. Vitreous hemorrhage (VH) is a complication observed in 12–19% of cases [6], intraocular foreign bodies (IOFBs) can exhibit a incidence from 6% to 42% in open globe injuries (OGIs) [7,8,9,10], and endophthalmitis, a severe inflammatory condition, may potentially complicate the OGI in up to 17% of cases [11,12]. The current management strategy in traumatic lens subluxation and dislocation is pars plana vitrectomy (PPV) combined with lensectomy, which provides the possibility to remove the lens as well as treat posterior segment complications [13]. In this study, we retrospectively evaluated the files of all patients with lens dislocation or subluxation that underwent PPV and lensectomy over an 11-year period with the aim to describe the clinical features, surgical outcomes and risk factors associated with poor prognosis.

## 2. Materials and Methods

### 2.1. Study Design and Subjects

This is a retrospective single-center study carried out on the medical files of 70 patients who underwent PPV–lensectomy for the treatment of traumatic lens dislocation or subluxation between the 1 January 2010 and the 31 December 2020 at the Department of Ophthalmology, Emergency County Hospital, “Iuliu Hațieganu” University of Medicine and Pharmacy from Cluj-Napoca, Romania. The study was approved by The Ethics Committee belonging to the “Iuliu Hațieganu” University of Medicine and Pharmacy (number 273/10 May 2023) and the Institutional Review Board of Cluj County Emergency Clinical Hospital (number 35/16 February 2023) and was conducted in accordance with the Declaration of Helsinki. Informed consent was obtained from each patient prior to surgery.

Using medical files, data were extracted from a series of consecutive eye trauma cases that were admitted and managed by a single surgeon (SDN) over an 11-year timeframe. Among 206 ocular trauma cases that underwent PPV during this specified timeframe, a total of 70 cases (34%) exhibited lens dislocation and subluxation and were included in this study. The inclusion criteria were the following: all cases of complete lens dislocation, lens subluxation or dislocated lens fragments in the vitreous cavity which occurred as a result of ocular trauma and were treated via a posterior approach with PPV and lensectomy. We excluded all patients with traumatic lens subluxations which could be managed by phacoemulsification, as well as patients in which intravitreal luxation of the lens or lens fragments occurred as a complication of cataract surgery and not ocular trauma.

A comprehensive ophthalmological examination was performed in all cases and the following data were obtained from patients’ records: age, gender, setting and type of injury, initial and final best corrected visual acuity (BCVA) (Snellen Chart), and co-existing trauma-related findings such as hyphema, iridodialysis, secondary glaucoma, VH and retinal detachment (RD). If the light-conducting media were opacified by hemorrhage or other ocular lesion and the fundus could not be evaluated clinically, B-mode ultrasonography was used to assess the status of the retina. In all cases with concurrent IOFB, the diagnosis was established with orbital computed tomography (CT) and information regarding the location, material and size were collected.

All study data were documented within an Excel spreadsheet. The medical charts were reviewed by the study coordinator, who ensured their accuracy and reliability. In cases of missing data, information was obtained from the attending surgeon. The data forms were subsequently sent to the study statistician and in cases involving data errors or inconsistencies, the coordinator addressed and corrected all data discrepancies. 

### 2.2. Surgical Technique

The goal of surgery was to remove the lens and restore vision as much as possible. In cases of OGIs with leaking wounds, a primary suture was performed at the beginning of the surgery to restore the structural integrity of the globe. 

**Figure 1:**
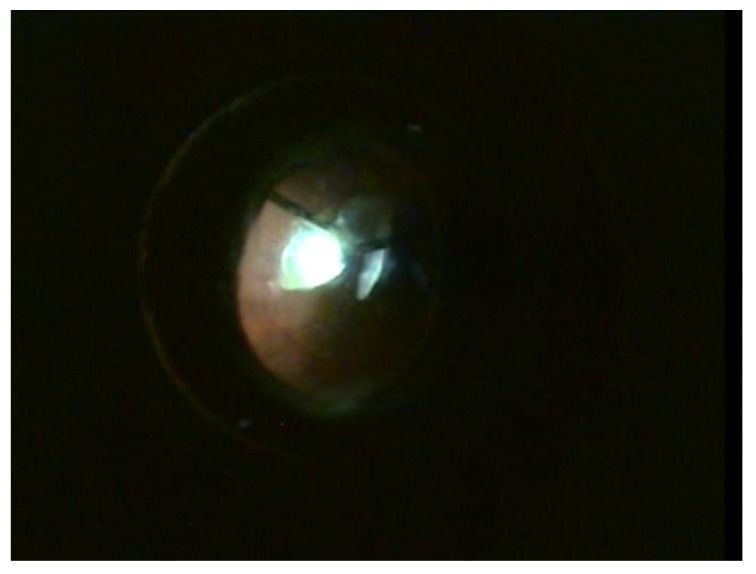
Extraction of lens fragments luxated in the vitreous cavity with the vitrector.

**Figure 2:**
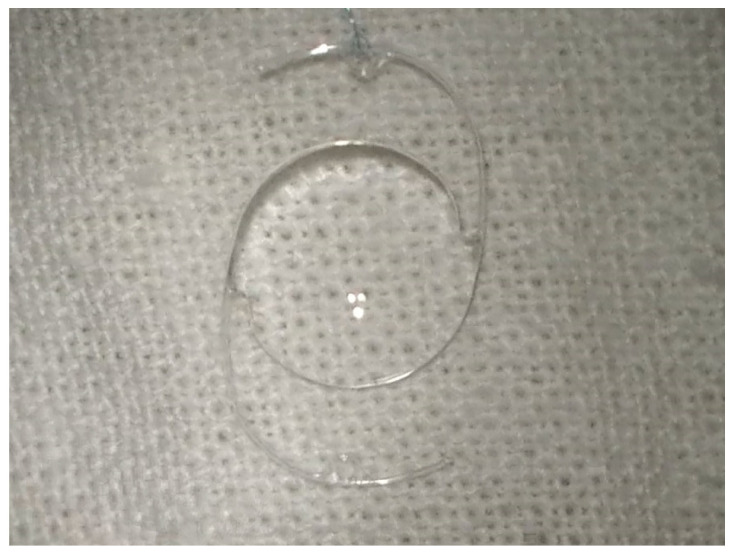
Rigid lens implant designed for being sutured at the sclera.

**Figure 3:**
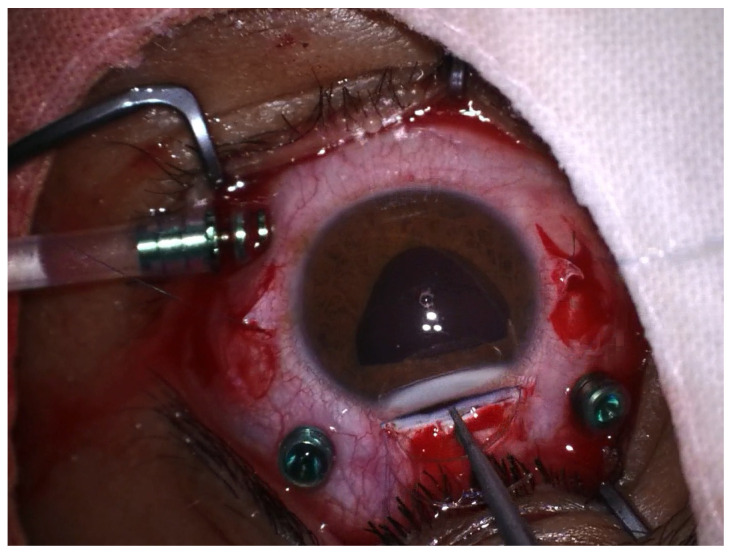
Insertion of the IOL.

**Figure 4:**
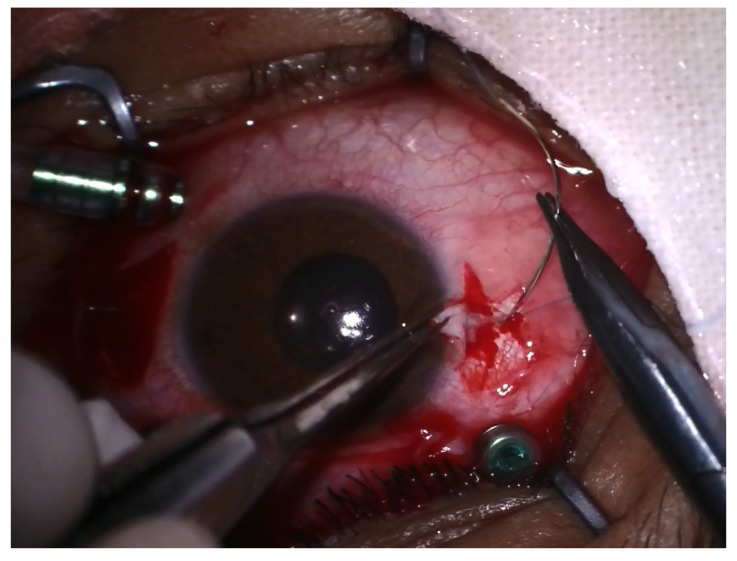
Scleral suturing of the IOL.

Surgical intervention was carried out in peribulbar anesthesia, with the exception of 4 pediatric patients who underwent general anesthesia. A standard 23-G vitrectomy technique was carried out. Three sclerotomies 3.5 mm away from the limbus, two at the 10 and 2 o’clock positions and one infero-temporally for the infusion line, were performed. First, vitreous adhesions around the lens were eliminated to minimize vitreous traction during manipulation; then, the vitrector was used to lift the lens from the surface of the retina into the middle or anterior vitreous cavity. The lens was removed either with the vitrector as shown in Figure 1 or with the fragmatome, depending on its hardness. In cases with posterior capsular rupture and lens fragments dislocated anteriorly and posteriorly, irrigation–aspiration and vitrectomy cutters were used to remove them, respectively. The preservation of the capsular bag was intended wherever possible. After successful removal of the lens, a posterior chamber intraocular lens implant (IOL) was inserted at the end of vitrectomy or at a later stage. A rigid polymethylmethacrylate (PMMA) IOL was used for scleral fixation, as seen in Figure 2, and an acrylic foldable monofocal IOL was used for cases with capsular support. The decision on the appropriate technique to place the IOL was made on a case-by-case basis. The ab interno method of scleral fixation of the IOL was performed in cases with a lack of an optimal support bag. Following vitrectomy, limbal peritomy to expose the sclera was performed and two flaps of one-third scleral thickness at 3 and 9 o’clock were created. Subsequently, a 4 mm superior corneoscleral tunnel was carried out Figure 3. Double-armed 10.0 prolene sutures were tied to each IOL haptic; then, both needles were inserted through the corneoscleral tunnel, under the iris, to exit at the level of the scleral flap, approximately 1.5 mm away from the limbus. Next, the IOL was placed in the posterior chamber and the sutures were tied under the scleral flaps, as seen in Figure 4. Conjunctival and corneoscleral incisions were closed with 7.0 vicryl and 10.0 nylon, respectively. In cases with mild capsular instability, a foldable IOL was placed in the sulcus or in the capsular bag through a 2.2 mm superior corneal incision. 

Additional surgical procedures were performed when indicated. If present, RD was treated by fluid/air exchange, followed by laser or cryotherapy to seal the retinal tear and silicone oil tamponade; IOFBs were extracted with the help of an intraocular magnet after having eliminated all its adhesions with the surrounding structures. The supero-temporal sclerotomy was enlarged to allow the extraction of the IOFB according to its size. All cases were performed by the same experienced vitreoretinal surgeon (SDN).

### 2.3. Statistical Analysis

MedCalc^®^ Statistical Software version 20.104 (MedCalc Software Ltd., Ostend, Belgium; https://www.medcalc.org; accessed on 9 September 2023) was used to analyze the data. Categorical variables were expressed as frequency and percentage and numerical data as the median (minimum-maximum) or as the mean ± standard deviation. The outcome was evaluated according to the final BCVA, measured with a Snellen Chart, which was defined as poor <0.1 or good ≥0.1 (equal to 1/10 in decimal fraction). In comparative statistical analysis, categorical data were assessed by the Chi-square test or Fisher’s test. Univariate logistics regression was applied to examine the associations between risk factors and final BCVA. Statistical significance was assumed at a *p* value less than 0.05.

## 3. Results

### 3.1. Demographic Results

During a period of 11 years, 70 consecutive patients with lens dislocation or subluxation were treated by PPV–lensectomy in our department. The mean age of our study population was 57.9 ± 17.6 (range, 6–85) years. Among these, 21 cases (30%) were female and 49 (70%) were males. The right eye was injured in 37 (52.8%) cases and left eye in 33 (47.2%). Most traumas occurred during household activities and three cases (4.3%) were the result of physical assaults. No patient was wearing eye protection at the time of injury. Data related to patients’ demographic characteristics are presented in Table 1. No correlation was observed between demographic characteristics and final visual outcome.

### 3.2. Initial BCVA, Visual Outcome and Prognostic Factors

At presentation, from 70 patients, only 6 (8.6%) had BCVA ≥ 0.1, whereas in 64 of them (91.4%), BCVA was <0.1. After surgery, BCVA ≥ 0.1 was noted in 32 patients (45.71%). A total of 10 (14.3%) patients had a final BCVA of less than counting fingers (CF); of these, 5 cases developed retinal detachment (RD) with proliferative vitreoretinopathy, 1 case had IOFB with concurrent RD, 1 case developed end-stage secondary glaucoma, 1 case had macular necrosis and 2 cases remained aphakic because they refused IOL implantation. A summary of preoperative and postoperative BCVA is listed in Table 2. We found that BCVA at presentation was significantly correlated with final visual outcome throughout the study, *p* = 0.000.

Closed globe injury (CGI) was present in 49 cases (70.0%) and open globe injury (OGI) in 21 (30.0%) cases. The injury was located at the central cornea in eight cases (38.1%), peripheral cornea in six cases (28.6%) and sclera in seven cases (33.3%). A primary suture was carried out in our department in 14 cases (66.7%); of these, 12 cases (85.7%) had primary repair within 48 h from injury, of which 5 had lensectomy performed at the same time, and for 2 cases (14.3%), primary repair was carried out 3 and 7 days post-injury, respectively. Self-sealing wounds were noted in three cases in which lensectomy was performed 2 weeks, 2 months and 8 months post-injury, respectively. The other four cases were referred to our institution after having undergone primary wound repair in other facilities; in two of them, primary repair was performed within 24 h from injury and in the remaining two, the time to repair was not noted.

A summary of the prognostic factors is illustrated in Table 3. The mean duration from injury to surgery was 230.1 days (range, 3 h to 11 years; median, 14 days). This long interval also reflects the time from injury to presentation at our department. The time interval until the operation was established by the surgeon during the initial examination, based on the severity of co-existing injuries and the patient’s desire. The lens was removed within 1 month from trauma in 53 cases (75.7%), between 1 and 6 months in 9 cases (12.9%) and more than 6 months in 8 cases (11.4%). The timing of the surgical intervention did not correlate with the final visual outcome, *p* = 1.000.

The dislocation mechanism was zonular lysis in 59 cases (84.3%) and capsular rupture in 11 cases (15.7%). Among all 70 cases, 21 eyes (30.0%) exhibited lens subluxation, 38 (54.3%) presented complete zonular dialysis with lens dislocation and 11 (15.7%) had lens fragments in the anterior chamber and vitreous cavity due to capsular rupture. Lens subluxation was located anteriorly in 6 (8.6%) eyes and posteriorly in 15 of them (21.4%); dislocations were anterior in 3 cases (4.3%) and posterior in 35 cases (50.0%).

IOFBs were identified in 6 of the 21 patients with OGIs (28.6%). Capsular rupture with lens fragment migration was present in four patients and zonular lysis in two of them. IOFB was caused by hammering metal on metal in four patients and flying splinters after wood cutting in two patients. The length of the IOFB varied between 2 mm and 8 mm; three IOFBs were located in the vitreous cavity and three of them were embedded in the retina. The association of an IOFB did not result in a poorer visual outcome, *p* = 1.000; three patients within this category ended up with BCVA ≥ 0.1.

RD was identified in 13 patients (18.6%) and was one of the factors with a negative impact on the visual outcome, *p* = 0.014. Of these, seven RDs were diagnosed at presentation and six during follow-up. Among the seven RD cases diagnosed at presentation, none of them recovered useful vision; four exhibited extensive retinal fibrosis, two had concurrent IOFB and one remained aphakic due to the patient’s choice. In the group of patients with RD during follow-up, only two recovered BCVA ≥ 0.1; visual outcome was compromised for the remaining four: two of them with IOFBs at presentation and the other two with secondary glaucoma.

Iridodonesis was identified in 23 patients (32.9%), with all cases (100%) being the result of zonular injury. The majority of these cases fall within the CGI category, 22 (95.7%), and only 1 (4.3%) was the result of a corneal penetrating injury. Its presence was significantly associated with poorer visual outcome *p* = 0.011. Other variables such as phacodonesis, VH, hyphema and secondary glaucoma were analyzed but did not correlate with the final visual outcome.

Most of the cases in our study presented with extensive or complete zonular disruption and a lack of optimal capsular support for the intraocular implant (IOL); as a result, the predominantly used method for implantation was scleral fixation. The final status of the lens is illustrated in Table 4. A total of 51 patients (72.9%) underwent scleral IOL fixation surgery. Per primam implantation of the IOL was possible in 29 of these cases (56.9%), whereas the IOL was inserted at a later stage in 22 cases (43.1%). A total of 15 patients (21.4%) remained aphakic. The decision not to implant the IOL was based on the following data: the presence of extensive retinal fibrosis in six cases, macular necrosis in one case and advanced glaucoma with subtotal excavation of the optic nerve in one case; seven eyes remained aphakic due to the patient’s choice.

IOL power calculations were made according to the A-scan axial length and keratometry measurements of the injured eye when possible or of the fellow eye when injuries interfered with calculations.

Table 5 displays the comparative distribution of final VA between our study and the ocular trauma score (OTS). Notably, there was similarity in the distribution of the final VA with the OTS study for eyes within OTS category 4. While certain disparities existed among particular subgroups in comparison to the OTS, it can be generally concluded that a higher OTS was indicative of a more favorable prognostic outcome, with a total *p* value ≤0.001 in all OTS categories.

## 4. Discussion

Ocular trauma represents the primary cause of lens dislocations and subluxations [14]. Traumatic ectopia lentis usually occurs after a direct blow to the eye with a high-energy projectile, such as a baseball or golf ball, or after blunt trauma to the head [15]. The prevalence of traumatic ectopia lentis is unknown. According to the World Health Organization (WHO), ocular injuries account for 5–16% of ophthalmological visits [2], but lens dislocations and subluxations make up a low overall proportion of visits. During a period of 11 years, we managed 70 patients with ectopia lentis that required PPV–lensectomy, including cases of penetrating injuries with capsular rupture and the posterior migration of lens fragments. This represents 32.9% of the total eye injuries we treated with PPV during that period of time (213 cases).

The mean age in our population study was 57.9 ± 17.6 years with males being more affected. Chaudhry et al. [16] found similar age results, although the incidence of ocular trauma is known to be higher in young active subjects [17,18]. This finding suggests the lack of education regarding the protection against eye trauma, regardless of age. Also, greater attention should be paid to educating the population regarding the importance of emergency presentation to the ophthalmologist in the case of eye trauma; thus, the rate of ocular complications and the morbidity associated with eye trauma are reduced, in parallel with increasing the chances of preserving useful vision [19].

PPV combined with lensectomy represents the method of choice for the management of severe lens subluxations (180 degrees or more) and dislocations [13,16,20,21,22]. The combined surgical approach of pars plana vitrectomy (PPV) and lensectomy has been documented to offer numerous advantages. Eyes with posterior lens or lens fragment dislocations are particularly susceptible to vision-threatening complications, including RD, persistent uveitis, secondary glaucoma and cystoid macular edema [23]. It has been reported in the literature that PPV–lensectomy effectively reduces these complications in a significant proportion of affected patients [23]. This technique operates on the principle of minimizing vitreoretinal traction by eliminating adhesions between the lens and vitreous, as well as those between the vitreous and the retina. Ideally, posterior vitreous detachment is induced prior to any manipulation of the lens material; this will also minimize the risk of postoperative retinal breaks and RD [24,25]. Additionally, it is recognized that ocular trauma often accompanies complex injuries such as RD, VH, IOFBs and corneoscleral laceration. The PPV–lensectomy approach permits intraocular examination during surgery and can be combined with other surgical interventions, such as IOFB extractions, IOL implantation, cryoretinopexy and laser treatments, among others [26].

The timing of surgery has changed over the years. In the past, it was considered that posterior dislocation could be well tolerated for years and managed only with optical correction of aphakia, PPV being reserved only for symptomatic cases [13,27]. Nowadays, thanks to innovative techniques in vitreoretinal surgery, such cases can be solved more easily and safely. In addition, a series of recognized complications, such as phacoanaphylactic uveitis, phacolytic glaucoma and retinal damage due to mechanical friction, are avoided by the complete removal of the ectopic lens [28,29]. However, PPV in the context of a traumatized eye is challenging and optimal visualization is sometimes compromised due to anterior segment lesions. In order to have a better intraoperative visualization, some authors suggested that lensectomy should be delayed until the corneal lesions and edema subside [30]. In situations of capsular damage with lens migration in the anterior chamber or vitreous cavity, the likelihood of developing intraocular hypertension and inflammation is higher, as are the risks of intraoperative complications in the case of postponing the intervention; therefore, lensectomy in these circumstances is recommended early [30,31]. The timing of PPV varied widely in our study; the median time between the traumatic event and lens removal was 14 days (range, 3h—11 years). However, the timing of the lensectomy did not have an impact on the visual outcome in this study. There are studies with similar timings of surgery [16,32], but there are also studies in which lens removal was conducted at a median time of 60 months following the traumatic event [21]. This discrepancy may be due to the large variety in the clinical manifestations and severity of eye injuries, surgeon’s decisions and patient’s preferences.

After lens removal, visual rehabilitation requires the use of an IOL. Primary or secondary IOL implantation in the setting of ocular trauma remains controversial. On the one hand, primary IOL implantation yields potential advantages including the following: reducing the risk of developing lens-induced inflammation, restoring binocular vision, improving the recovery, reducing the potential risks related to anesthesia, and minimizing the total surgical trauma, cost and time of hospital admission [33,34,35,36]. In contrast, secondary IOL implantation at a later stage when acute trauma-related lesions have subsided provides a better visualization during surgery, better IOL calculation and the possibility of assessing the visual potential [37]. Studies showed that there is no difference in the visual prognosis in the case of primary vs. secondary IOL implantation [38,39]. In our study, the decision to implant a primary or a secondary IOL was based mainly on the status of the eye at the time of surgery and on surgeon’s preference and reasoning.

Ideally, an IOL is inserted into the capsular bag. In practice, the absence of capsular support or identification of extensive zonular lesions makes this technique impossible to perform and requires IOL implantation by alternative methods. The choice of IOL type has been a highly debated topic in the literature. Malbran et al. first described the method of transscleral IOL fixation and, since then, it has been widely adopted [40]; in the last decade, several sutureless scleral fixation techniques have been developed offering a faster and easier learning curve [41,42]. There are also other techniques for IOL placement, including its insertion into the anterior chamber, anterior or posterior iris-claw IOLs or it being sutured to the posterior iris. The use of scleral-sutured IOLs offers the advantage of lower complication rates, such as corneal endothelial decompensation, pupillary block or angle closure when compared to the other methods [43,44]; however, this surgery was found to be more technically demanding with a steep learning curve [38,45]. On the other hand, the anterior chamber IOL technique has been associated with fewer refractive errors due to standardized IOL power calculations, no suture-related issues, minimal conjunctival manipulation, more predictable IOL position and easier technique [46]. In the majority of cases, we opted for sutured scleral fixation of a rigid, one-piece posterior chamber PMMA IOL as the surgeon felt most comfortable with this technique. In addition, the safety and efficacy of sutured scleral fixation posterior chamber IOL has been proven over time, whereas sutureless techniques need more long-term results in order to be definitely validated. Other posterior chamber IOLs, such as iris-claw, have been associated with inferior decentration, as the technique requires more surgical skills, because the IOL haptic is not directly seen by the surgeon. In addition, the A-constant is not provided by the manufacturer and they limit the myosis and mydriasis of the pupil, making visualization difficult in the event of a subsequent PPV [47,48].

We analyzed several ocular trauma features and their predictive value for the final visual outcome. Traumatic eye injuries may lead to a variety of presenting signs and symptoms, as the nature of the injury is variable in each case. Because we are a tertiary care center, many of the patients had severe anterior and posterior segment injuries upon presentation, in addition to ectopia lentis. Many studies proved that presenting BCVA is one of the most important parameters in predicting the final outcome in OGIs [49,50]. However, little information is available in regard to this prognostic factor in traumatic lens subluxations and dislocations. Our results showed that initial BCVA correlated significantly with final BCVA, *p* = 0.000. An initial good BCVA reflects a milder ocular damage as compared to poor BCVA, which suggests more substantial damage to the ocular structures, such as RD or VH. Greven et al. found in their study that there was a trend for patients with good presenting BCVA to have a good postoperative BCVA, but it did not quite reach statistical significance, *p* = 0.078 [22]. A study conducted by Yasa et al. found that postoperative ambulatory vision is better in patients with a presenting BCVA of 5/200 or more, *p* = 0.036 [18]. Bielinski et al.’s study showed that the improvement of BCVA after surgery was statistically significant, *p* = 0.005; but unlike us, they used a sutureless method for scleral IOL fixation [51]. BCVA ≥ 0.1 was reached in 45.7% of cases in our study. Other studies described similar visual gains in traumatic lens dislocations and subluxations managed by PPV combined with lensectomy and scleral-sutured IOL [16], the poor postoperative BCVA being associated with posterior segment lesions [22]. The iris is a delicate structure which can display iridodonesis and iridodialysis as a result of blunt or penetrating eye trauma [52]. The incidence of iridodonesis among our patients was 32.9% and was associated with a poor visual outcome, *p* = 0.011. It was hypothesized that the iris is the first most affected ocular structure in trauma, followed by the lens [53]. To our knowledge, iridodonesis has not been found so far as a factor influencing the final BCVA; however, we consider that there is a direct relationship between these results and the association of posterior segment lesions; of the 23 cases with iridodonesis, 11 presented either RD, VH, choroidal rupture or secondary glaucoma. Complications such as VH, endophthalmitis, secondary glaucoma and hyphema may or may not accompany lens dislocations; however, their presence was not predictive for visual outcome, which is in accordance with other studies [18,22,54].

The visual outcome in patients with traumatic ectopia lentis can be severely affected if RD is associated [17], unlike those without posterior segment lesions [4]. On the one hand, blunt ocular trauma most often induces RD by increased traction on the vitreous base [55]; subluxation can also cause vitreous prolapse in the anterior chamber and its subsequent incarceration and tractions transmitted to the vitreous base which lead to the formation of retinal tears and RD [56]. In penetrating injuries, in addition to intraocular proliferation and retinal lesions produced by an IOFB or the trauma itself, the rupture of the lens capsule or the complete dislocation of the lens in the vitreous can lead to a reduction in the hyaluronan concentration within the vitreous body which accelerates the process of syneresis and decreases the shock-absorbing property, thus leading to an increase in the torsional forces transmitted to areas of vitreoretinal adhesions, subsequently increasing the risk for RD [57]. It was suggested that retinal tears and RD may occur after the scleral suturing of the IOL as a result of trauma to the vitreous base caused by the needle penetrating the eye wall or by its haptics [4]. However, this suggestion was contradicted by Lee et al. who found an incidence of RD after scleral suturing of the IOL of 4.9%, similar with reports not using scleral-fixated IOLs [58]. We found comparable results: within the group of 51 scleral-sutured IOLs in our study, RD occurred in 2 cases (3.9%). Overall, RD was one of the prognostic factors associated with a poor visual outcome in our study.

The OTS is a comprehensive scoring system that incorporates initial VA, the extent of ocular trauma and the presence or absence of elements such as globe rupture, endophthalmitis, RD and relative afferent pupillary defect (RAPD). In general, a higher OTS score tends to be indicative of a more favorable prognosis [59]. Numerous studies have assessed the predictability of the OTS. For instance, one study found that OTS can offer prognostic information in OGIs caused by deadly weapons [60]. Moreover, OTS was a useful indicator for final VA in cases of OGIs with concurrent IOFBs [61] and also in OGIs in children [62]. The predictability of OTS in traumatic cataracts in adults was evaluated by Ying et al. and it was found that OTS has high sensitivity and specificity for predicting the visual outcome of traumatic cataract patients in long-term follow-up [63]. Similarly, our research demonstrated the predictive capacity of OTS in relation to final VA outcomes in traumatic cataracts.

Our research has certain limitations, with the most notable among them being its retrospective nature and the relatively small sample size. Therefore, we cannot affirm with certainty that the factors analyzed in this case series that did not correlate statistically with the final visual outcome cannot also affect vision. An additional constraint is potential bias in data collection and the inability to control variables. In order to overcome this, all data in our study were collected by the same person; anonymization and de-identification was performed before analysis and all PPVs were performed by the same surgeon using similar techniques. The potential for selection bias exists because of our status as a tertiary care center, raising the likelihood of socioeconomic and referral biases being present. Lastly, an additional limitation lies in the absence of novel scleral fixation techniques or future perspectives. Our main goal in this study was to evaluate the final visual outcome for traumatic lens dislocation and to describe the technique we used. Although there are many scleral fixation techniques at the moment, we consider that the best technique is the one you feel most comfortable with. As our study is a retrospective one, some techniques described today did not exist at that time; each new technique used requires a learning curve and we consider that a sufficient patient volume is needed to make it worth learning different techniques. A report by the American Academy of Ophthalmology which analyzed 45 different studies described that no technique of scleral-fixing IOLs is superior to another [64]. We also believe that there is no ideal IOL fixation procedure and that it is best to find, master and stick with a technique you feel comfortable performing. Nonetheless, we consider that the study highlighted the positive result of PPV combined with lensectomy in traumatic lens subluxations and dislocations.

## 5. Conclusions

This study presents the surgical outcomes of PPV combined with lensectomy in traumatic ectopia lentis in a tertiary care center. The factors that predicted the poor visual outcome in our series were poor BCVA at presentation (*p* = 0.000), RD (*p* = 0.014) and iridodonesis (0.011). PPV combined with lensectomy followed by primary or secondary IOL implantation provided a good BCVA ≥ 0.1 in 45.71% of eyes. The suture of the IOL at the sclera was a safe and effective method to correct aphakia following traumatic ectopia lentis.

## Figures and Tables

**Table 1:** Demographic characteristics.

Variable		BCVA < 0.1	BCVA ≥ 0.1	*p* Value
Gender	Female (n = 21) Male (n = 49)	11 (28.9%) 27 (71.1%)	10 (31.3%) 22 (68.7%)	1.000
Age	<50 (n = 18) ≥50 (n = 52)	10 (26.3%) 28 (73.7%)	8 (25.0%) 24 (75.0%)	1.000
Eye	Right (n = 37) Left (n = 33)	20 (52.6%) 18 (47.4%)	17 (53.1%) 15 (46.9%)	1.000
Eye protection	Yes (n = 0) No (n = 70)	- 38 (100%)	- 32 (100%)	
Location	Rural (n = 46) Urban (n = 24)	27 (71.1%) 11 (28.9%)	19 (59.4%) 13 (40.6%)	0.440

BCVA: best corrected visual acuity.

**Table 2:** Visual outcome in patients with traumatic lens dislocation.

VA	Preoperative BCVA n (%)	Postoperative BCVA n (%)
≥0.1	6 (8.57)	32 (45.71)
<0.1	18 (25.71)	19 (27.14)
CF	20 (28.57)	9 (12.85)
HM	20 (28.57)	5 (7.14)
LP	4 (5.71)	2 (2.85)
NLP	2 (2.85)	3 (4.28)
Total	70	70

VA: visual acuity; BCVA: best corrected visual acuity; CF: counting fingers; HM: hand motion; LP: light perception; NLP: no light perception.

**Table 3:** Prognostic factors.

Factor	Final VA, n (%)		*p* Value
	<0.1	≥0.1	
Age			
<50	10 (26.3)	8 (25.0)	1.000
≥50	28 (73.7)	24 (75.0)	
Initial VA			
<0.1	37 (97.4)	26 (81.3)	**0.000**
≥0.1	1 (2.6)	6 (18.7)	
Iridodonesis			
Yes	7 (18.4)	16 (50.0)	**0.011**
No	31 (81.6)	16 (50.0)	
Phacodonesis			
Yes	2 (5.3)	5 (15.6)	0.234
No	36 (94.7)	27 (84.4)	
RD			
Yes	11 (28.9)	2 (6.2)	**0.014**
No	27 (71.1)	30 (93.8)	
IOFB			
Yes	3 (7.9)	3 (9.4)	1.000
No	35 (92.1)	29 (90.6)	
VH			
Yes	8 (21.1)	10 (31.3)	0.485
No	30 (78.9)	22 (68.7)	
Secondary glaucoma			
Yes	13 (34.2)	4 (12.5)	0.067
No	25 (65.8)	28 (87.5)	
Dislocation mechanism			
PC rupture	7 (18.4)	4 (12.5)	0.727
Zonular rupture	31 (81.6)	28 (87.5)	
Hyphema			
Yes	6 (15.8)	4 (12.5)	0.745
No	32 (84.2)	28 (87.5)	
Timing of PPV			
<1 month	29 (76.3)	24 (75.0)	1.000
>1 month	9 (23.7)	8 (25.0)	

n: number; VA: visual acuity; RD: retinal detachment; IOFB: intraocular foreign body; VH: vitreous hemorrhage; PC: posterior capsule; PPV: pars plana vitrectomy; Bold: *p* value was significant.

**Table 4:** Final status of the lens.

Final Lens Position	Number (%)
PC IOL scleral fixation	51 (72.9%)
PC IOL in sulcus	1 (1.4%)
PC IOL in the bag	3 (4.3%)
Aphakia	15 (21.4%)

PC IOL: posterior chamber intraocular lens.

**Table 5:** Final VA outcomes based on ocular trauma score.

OTS		Final VA Group										
		NLP		HM/LP		CF-<0.1		0.1–0.4		≥0.5		
	OTS	(%)	*p* Value	(%)	*p* Value	(%)	*p* Value	(%)	*p* Value	(%)	*p* Value	Total *p* Value
	Our Study (70)	N (%)		N (%)		N (%)		N (%)		N (%)		
1	OTS Our Study (2)	(73) 0 (0)	0.02	(17) 2 (100)	0.002	(7) 0 (0)	0.69	(2) 0 (0)	0.83	(1) 0 (0)	0.88	<0.001
2	OTS Our Study (16)	(28) 3 (18.75)	0.43	(26) 3 (18.75)	0.53	(18) 7 (43.75)	0.02	(13) 2 (12.5)	0.95	(15) 1 (6.25)	0.34	0.001
3	OTS Our Study (47)	(2) 0 (0)	0.32	(11) 2 (4.25)	0.18	(15) 22 (46.81)	<0.001	(28) 18 (38.3)	0.2	(44) 5 (10.64)	<0.001	<0.001
4	OTS Our Study (5)	(1) 0 (0)	0.82	(2) 0 (0)	0.74	(2) 0 (0)	0.74	(21) 0 (0)	0.25	(74) 5 (100)	0.18	<0.001
5	OTS Our Study	(0) 0 (0)	-	(1) 0 (0)	-	(2) 0 (0)	-	(5) 0 (0)	-	(92) 0 (0)	-	-

OTS: ocular trauma score; VA: visual acuity; NLP: no light perception; HM: hand motion; LP: light perception; CF: counting fingers; N: number.

## Data Availability

The data presented in this study are available on request to the corresponding author.

## References

[1] Klopfer J., Tielsch J.M., Vitale S., See L.C., Canner J.K. (1992). Ocular Trauma in the United States: Eye Injuries Resulting in Hospitalization, 1984 through 1987. Arch. Ophthalmol..

[2] Négrel A.D., Thylefors B. (1998). The Global Impact of Eye Injuries. Ophthalmic Epidemiol..

[3] Bord S.P., Linden J. (2008). Trauma to the Globe and Orbit. Emerg. Med. Clin. N. Am..

[4] Salehi-Had H., Turalba A. (2010). Management of Traumaticcrystalline Lens Subluxation and Dislocation. Int. Ophthalmol. Clin..

[5] Kuhn F., Morris R., Witherspoon C.D., Mann L. (2007). Blunt-Force Injuries Involving the Posterior Segment. Retin. Physician.

[6] Spraul C.W., Grossniklaus H.E. (1997). Vitreous Hemorrhage. Surv. Ophthalmol..

[7] Nicoară S.D., Irimescu I., Călinici T., Cristian C. (2015). Intraocular Foreign Bodies Extracted by Pars Plana Vitrectomy: Clinical Characteristics, Management, Outcomes and Prognostic Factors. BMC Ophthalmol..

[8] Hapca M.C., Muntean G.A., Drăgan I.A.N., Vesa Ș.C., Nicoară S.D. (2022). Outcomes and Prognostic Factors Following Pars Plana Vitrectomy for Intraocular Foreign Bodies—11-Year Retrospective Analysis in a Tertiary Care Center. J. Clin. Med..

[9] Yeh S., Colyer M.H., Weichel E.D. (2008). Current Trends in the Management of Intraocular Foreign Bodies. Curr. Opin. Ophthalmol..

[10] Liu C.C.H., Tong J.M.K., Li P.S.H., Li K.K.W. (2017). Epidemiology and Clinical Outcome of Intraocular Foreign Bodies in Hong Kong: A 13-Year Review. Int. Ophthalmol..

[11] Gokce G., Sobaci G., Ozgonul C. (2015). Post-Traumatic Endophthalmitis: A Mini-Review. Semin. Ophthalmol..

[12] Hapca M.C., Vesa Ș.C., Nicoară S.D. (2023). Visual Outcomes and Prognostic Factors of Traumatic Endophthalmitis Treated by Pars Plana Vitrectomy: 11 Years Retrospective Analysis. J. Clin. Med..

[13] Marcus D.M., Topping T.M., Frederick A.R. (1995). Vitreoretinal Management of Traumatic Dislocation of the Crystalline Lens. Int. Ophthalmol. Clin..

[14] Jarrett WH I.I. (1967). Dislocation of the Lens. A Study of 166 Hospitalized Cases. Arch. Ophthalmol..

[15] Robinson D.H., Leonard B.N., Scott E.O. (2005). Harley’s Pediatric Ophthalmology.

[16] Chaudhry N.A., Belfort A., Flynn H.W.J., Tabandeh H., Smiddy W.E., Murray T.G. (1999). Combined Lensectomy, Vitrectomy and Scleral Fixation of Intraocular Lens Implant after Closed-Globe Injury. Ophthalmic Surg. Lasers.

[17] Kuhn F., Morris R., Witherspoon C.D., Mann L. (2006). Epidemiology of Blinding Trauma in the United States Eye Injury Registry. Ophthalmic Epidemiol..

[18] Yaşa D. (2017). Pars Plana Lensectomy Combined with Pars Plana Vitrectomy in Traumatic Ectopia Lentis. Beyoglu Eye J..

[19] Singhal A., Sharma A., Sasirekha M. (2020). Visual Outcome in Patients Operated for Traumatic Cataract: A Prospective Hospital Based Study. Indian J. Clin. Exp. Ophthalmol..

[20] Peyman G.A., Raichand M., Goldberg M.F., Ritacca D. (1979). Management of Subluxated and Dislocated Lenses with the Vitrophage. Br. J. Ophthalmol..

[21] Kodjikian L., Beby F., Spire M., Gambrelle J., Hubert I., Burillon C., Grange J.D., Garweg J.G. (2006). Combined Pars Plana Phacofragmentation, Vitrectomy, and Artisan Lens Implantation for Traumatic Subluxated Cataracts. Retina.

[22] Greven C.M., Collins A.S., Slusher M.M., Weaver R.G. (2002). Visual Results, Prognostic Indicators, and Posterior Segment Findings Following Surgery for Cataract/Lens Subluxation-Dislocation Secondary to Ocular Contusion Injuries. Retina.

[23] Moore J.K., Scott I.U., Flynn H.W., Smiddy W.E., Murray T.G., Kim J.E., Vilar N.F., Pereira M.B., Jorge R. (2003). Retinal Detachment in Eyes Undergoing Pars Plana Vitrectomy for Removal of Retained Lens Fragments. Ophthalmology.

[24] Kazemi S., Wirostko W.J., Sinha S., Mieler W.F., Koenig S.B., Sheth B.P. (2000). Combined Pars Plana Lensectomy-Vitrectomy with Open-Loop Flexible Anterior Chamber Intraocular Lens (AC IOL) Implantation for Subluxated Lenses. Trans. Am. Ophthalmol. Soc..

[25] Oh J., Smiddy W.E. (2010). Pars Plana Lensectomy Combined with Pars Plana Vitrectomy for Dislocated Cataract. J. Cataract Refract. Surg..

[26] Girard L.J. (1982). Pars Plana Lensectomy by Ultrasonic Fragmentation. Surv. Ophthalmol..

[27] Seo M.S., Yoon K.C., Lee C.H. (2002). Phacofragmentation for the Treatment of a Completely Posterior Dislocation of the Total Crystalline Lens. Korean J. Ophthalmol..

[28] Jurman M., Jones W.L., Harris S.L. (1990). Traumatic Dislocation of the Crystalline Lens with Delayed Total Retinal Detachment. J. Am. Optom. Assoc..

[29] Zhang H., Dong J., Jin K., Wang G., Xu D., Huo M. (2012). Efficacy of Removing Dislocated Lens Using Intravitreal Phacoemulsification. Eye Sci..

[30] Tabatabaei S.A., Rajabi M.B., Tabatabaei S.M., Soleimani M., Rahimi F., Yaseri M. (2017). Early versus Late Traumatic Cataract Surgery and Intraocular Lens Implantation. Eye.

[31] Agrawal R., Keane P.A., Singh J., Saihan Z., Kontos A., Pavesio C.E. (2016). Classification of Semi-Automated Flare Readings Using the Kowa FM 700 Laser Cell Flare Meter in Patients with Uveitis. Acta Ophthalmol..

[32] Slusher M.M., Greven C.M., Yu D.D. (1992). Posterior Chamber Intraocular Lens Implantation Combined with Lensectomy-Vitrectomy and Intraretinal Foreign-Body Removal. Arch. Ophthalmol..

[33] Rubsamen P.E., Irvine W.D., McCuen B.W., Smiddy W.E., Bowman C.B. (1995). Primary Intraocular Lens Implantation in the Setting of Penetrating Ocular Trauma. Ophthalmology.

[34] Lam D.S.C., Tham C.C.Y., Kwok A.K.H., Gopal L. (1998). Combined Phacoemulsification, Pars Plana Vitrectomy, Removal of Intraocular Foreign Body (IOFB), and Primary Intraocular Lens Implantation for Patients with IOFB and Traumatic Cataract. Eye.

[35] Muga R., Maul E. (1978). The Management of Lens Damage in Perforating Corneal Lacerations. Br. J. Ophthalmol..

[36] Andenmatten R., Gonvers M. (1993). Sophisticated Vitreoretinal Surgery in Patients with a Healthy Fellow Eye—An 11-Year Retrospective Study. Graefe’s Arch. Clin. Exp. Ophthalmol..

[37] Assi A., Chacra C.B., Cherfan G. (2008). Combined Lensectomy, Vitrectomy, and Primary Intraocular Lens Implantation in Patients with Traumatic Eye Injury. Int. Ophthalmol..

[38] Yalniz-Akkaya Z., Burcu A., Uney G.O., Abay I., Eksioglu U., Acar M.A., Ornek F. (2014). Primary and Secondary Implantation of Scleral-Fixated Posterior Chamber Intraocular Lenses in Adult Patients. Middle East Afr. J. Ophthalmol..

[39] Lee V.Y.W., Yuen H.K.L., Kwok A.K.H. (2003). Comparison of Outcomes of Primary and Secondary Implantation of Scleral Fixated Posterior Chamber Intraocular Lens. Br. J. Ophthalmol..

[40] Malbran E.S., Malbran E.J., Negri I. (1986). Lens Guide Suture for Transport and Fixation in Secondary IOL Implantation after Intracapsular Extraction. Int. Ophthalmol..

[41] Kumar D.A., Agarwal A., Packiyalakshmi S., Jacob S., Agarwal A. (2013). Complications and Visual Outcomes after Glued Foldable Intraocular Lens Implantation in Eyes with Inadequate Capsules. J. Cataract Refract. Surg..

[42] Yamane S., Sato S., Maruyama-Inoue M., Kadonosono K. (2017). Flanged Intrascleral Intraocular Lens Fixation with Double-Needle Technique. Ophthalmology.

[43] Wagoner M.D., Cox T.A., Ariyasu R.G., Jacobs D.S., Karp C.L. (2003). Intraocular Lens Implantation in the Absence of Capsular Support: A Report by the American Academy of Ophthalmology. Ophthalmology.

[44] Jacob S., Kumar D.A., Rao N.K. (2020). Scleral Fixation of Intraocular Lenses. Curr. Opin. Ophthalmol..

[45] Kjeka O., Bohnstedt J., Meberg K., Seland J.H. (2008). Implantation of Scleral-Fixated Posterior Chamber Intraocular Lenses in Adults. Acta Ophthalmol..

[46] Tsatsos M., Vartsakis G., Athanasiadis I., Papavasileiou E., Yesilirmak N., Ziakas N. (2022). Intraocular Lens Implantation in the Absence of Capsular Support: Iris Fixation. Eye.

[47] Calzetti G., Bellucci C., Tedesco S.A., Rossi M., Gandolfi S., Mora P. (2022). Tilt and Decentration of Posterior and Anterior Iris-Claw Intraocular Lenses: A Pilot Study Using Anterior Segment Optical Coherence Tomography. BMC Ophthalmol..

[48] Bellucci C., Perrella A., Rossi M., Papapicco A., Spadini F., Tedesco S.A., Gandolfi S., Mora P. (2023). Light- and Drug-Induced Pupillary Dynamics in Eyes with a Retropupillary Iris-Claw Intraocular Lens. Graefe’s Arch. Clin. Exp. Ophthalmol..

[49] Fujikawa A., Mohamed Y.H., Kinoshita H., Matsumoto M., Uematsu M., Tsuiki E., Suzuma K., Kitaoka T. (2018). Visual Outcomes and Prognostic Factors in Open-Globe Injuries. BMC Ophthalmol..

[50] Rofail M., Lee G.A., O’rourke P. (2006). Prognostic Indicators for Open Globe Injury. Clin. Exp. Ophthalmol..

[51] Bieliński P., Jasielska M., Wyszyńska A., Winiarczyk M., Mackiewicz J. (2019). Pars Plana Vitrectomy with Transscleral Fixation of Posterior Chamber Lens in the Treatment of Post-Traumatic Lens Dislocation. Int. Ophthalmol..

[52] Mayer C.S., Hoffmann A.M., Prahs P., Reznicek L., Khoramnia R. (2020). Functional Outcomes after Combined Iris and Intraocular Lens Implantation in Various Iris and Lens Defects. BMC Ophthalmol..

[53] Duke-Elder S. (1954). Text-Book of Ophthalmology.

[54] Huang H.M., Kao M.L., Kuo H.K., Tsai S.H., Chen Y.J., Liu C.C. (2004). Visual Results and Complications after Trans Pars Plana Vitrectomy and Lensectomy for Lens Dislocation. Chang Gung Med. J..

[55] Cox M.S. (1980). Retinal Breaks Caused by Blunt Nonperforating Trauma at the Point of Impact. Trans. Am. Ophthalmol. Soc..

[56] Ke G., Zhou E., Zhu K., Wei Y., Wang Z., Jia Y., Wang S., Gu Y. (2020). Retinal Break Associated with Traumatic Lens Dislocation or Subluxation Requiring Vitrectomy. Graefe’s Arch. Clin. Exp. Ophthalmol..

[57] Ghazi N.G., Green W.R. (2002). Pathology and Pathogenesis of Retinal Detachment. Eye.

[58] Lee J., Lee J., Chung H. (1998). Factors Contributing to Retinal Detachment after Transscleral Fixation of Posterior Chamber Intraocular Lenses. J. Cataract Refract. Surg..

[59] Kuhn F., Maisiak R., Mann L., Mester V., Morris R., Witherspoon C. (2002). The Ocular Trauma Score (OTS). Ophthalmol. Clin. N. Am..

[60] Sobacı G., Akin T., Erdem Ü., Uysal Y., Karagül S. (2006). Ocular Trauma Score in Deadly Weapon–Related Open-Globe Injuries. Am. J. Ophthalmol..

[61] Unal M.H., Aydin A., Sonmez M., Ayata A., Ersanli D. (2008). Validation of the Ocular Trauma Score for Intraocular Foreign Bodies in Deadly Weapon-Related Open-Globe Injuries. Ophthalmic Surg. Lasers Imaging Retin..

[62] Uysal Y., Mutlu F.M., Sobac G. (2008). Ocular Trauma Score in Childhood Open-Globe Injuries. J. Trauma Acute Care Surg..

[63] Qi Y., Zhang Y.F., Zhu Y., Wan M.G., Du S.S., Yue Z.Z. (2016). Prognostic Factors for Visual Outcome in Traumatic Cataract Patients. J. Ophthalmol..

[64] Shen J.F., Deng S., Hammersmith K.M., Kuo A.N., Li J.Y., Weikert M.P., Shtein R.M. (2020). Intraocular Lens Implantation in the Absence of Zonular Support: An Outcomes and Safety Update. Ophthalmology.

